# How Important Is Death?

**DOI:** 10.1016/j.shj.2024.100396

**Published:** 2024-12-20

**Authors:** Anthony DeMaria

**Affiliations:** University of California, San Diego, La Jolla, California, USA

Contemporary clinical trials increasingly employ a composite endpoint. In fact, it is difficult to find an impactful cardiovascular trial that has not had composite endpoints. The rationale for such endpoints is both that doing so increases events and thus reduces the sample size necessary for significance, and that intervention might be of value by decreasing only some events and not others. The major limitation of composite endpoints is that individual events are almost never of the same importance. Although some things might be worse than death, obviously death is a much more severe outcome than, for instance, recurrent hospitalization or repeat intervention. Despite this limitation, the use of composite endpoints tends to be the rule rather than the exception in cardiovascular trials.

The prevalence of composite events as the endpoint of clinical trials begets the question of how important death is compared to less severe events relative to risk. Stated another way, if an intervention did not increase survival, but rather changed the likelihood for a morbid event or offered the possibility of an increased quality of life, would the risk benefit ratio justify the intervention. The relevance of this consideration was highlighted by two recent trials that have the potential to alter guidelines and clinical practice in a very major way.

The EARLY Transcatheter Aortic-Valve Replacement for Asymptomatic Severe Aortic Stenosis (TAVR) Trial^1^ sought to answer the question of the optimal time for intervention in patients with severe aortic stenosis (AS). This is a question that has been unanswered but vigorously debated since I was an intern, and indeed could be applied to virtually all heart valve disease. As is probably known to all *Structural Heart* readers, the study enrolled 900 patients with severe but asymptomatic AS into subgroups receiving TAVR and controls undergoing surveillance. The primary endpoint was a composite of death, unplanned hospitalization, and stroke. The results demonstrated a significant benefit for TAVR, with a primary endpoint that occurred in 45% of the surveillance group vs. 27% of the TAVR group yielding a hazard ratio, 0.50. However, there was no significant difference between the groups in death or stroke. The benefit was nearly entirely due to a reduction in unplanned hospitalization in the surveillance cohort driven primarily by the progression of symptoms rather than acute events.

A brief consideration of the results would suggest that early TAVR should be strongly considered and even recommended in most patients with asymptomatic AS who fulfilled the entry criteria of the trial. As is true of most trials, this study had a few limitations, most prominently the lack of blinding. However, for this editorial, I would like to focus on the lack of benefit of the hard endpoints of stroke, and especially death. While TAVR has proven to be quite safe in this study and others, the occurance of few events is not the same as no events. Every invasive procedure involves some risk, and, as the saying goes, pacemaker implantation is a minor procedure when performed on someone else. In addition, TAVR trades one imperfect valve for another. Accordingly, I suspect some patients might view the data and conclude that they would rather pass on an invasive procedure, possible pacemaker, and dealing with a prosthetic valve and antithrombotic therapy. They might reason that, since they are not at risk of any hard endpoint, they would rather postpone an intervention until symptoms or some other accepted indication. Obviously, the consideration would be markedly different had death had been delayed by TAVR.

The TRILUMINATE Transcatheter Tricuspid Repair Trial (TRILUMINATE Trial)^2^ tested the hypothesis that transcatheter edge to edge repair (TEER) of the tricuspid valve would be of greater benefit than maximal medical therapy in patients with severe regurgitation. The investigators enrolled 370 patients randomly assigned to tricuspid TEER or medical therapy. The primary end point was a hierarchical composite (win ratio) that included death from any cause, tricuspid-valve surgery, hospitalization for heart failure, and an improvement in quality of life as measured with the Kansas City Cardiomyopathy Questionnaire (defined as an increase of at least 15 points). The win ratio for the primary endpoint demonstrated a benefit for the TEER group (1.48 vs. 1.06; *p* = 0.02). As with EARLY TAVR, there was no significant difference between cohorts in the hard endpoints of death, tricuspid surgery, or heart failure hospitalization. The superiority of TEER was due to the change in subjective evaluation of quality of life. The trial had limitations related to the difficulty in quantitatively assessing the magnitude of symptoms and tricuspid regurgitation, but most importantly the lack of blinding. However, as with EARLY TAVR, it was the lack of benefit on death and other hard outcomes that I believe might substantially alter the way the findings were analyzed by patients and physicians. All potential risks entailed in TAVR except pacemakers would apply to tricuspid TEER, and for the possible benefit of an increase in quality life observed in 50% of patients with TEER vs. 26% in controls. Again, TRILUMINATE raises relevant considerations regarding risk benefit ratio.

The purpose of this editorial is not to critique the 2 trials referred to. Both were well conducted, serious efforts to answer questions of great clinical importance. There is no doubt that they have greatly informed our thinking and approach to these common heart valve disorders. Rather, the purpose has been to demonstrate how patients and physicians may view the results differently than the conclusions might suggest.

In the final analysis, the assessment of risk-benefit lies in the hands of the beholder. Individual patients and physicians will view the same data and come to different conclusions. I have had patients who could not withstand the thought of having a bodily imperfection and sought an intervention even when not deemed necessary. Conversely, I have had patients who have indicated that “when my time’s up, it’s up,” and left it in the hands of fate even when an intervention was strongly justified and recommended. I personally do not think that the results are compelling enough for inclusion in guideline documents, but only time will tell. So, I anticipate that the way in which the results of EARLY TAVR and TRILUMINATE will be acted upon will be very variable. Clearly, the data will provide the basis for performing transcatheter intervention in a much larger population and earlier. It goes without saying that long term data may well document a robust benefit not only on quality of life but also on hard endpoints. Until that time, the clinical translation of EARLY TAVR and TRILUMINATE is likely to depend upon the importance patients and physicians place upon death and, to a lesser extent, other hard outcomes.

## Funding

The author has no funding to report.

## Disclosure Statement

The author reports no conflict of interest.
